# A bibliometric analysis of the studies in high-altitude induced sleep disturbances and cognitive impairment research

**DOI:** 10.3389/fphys.2023.1133059

**Published:** 2023-01-13

**Authors:** Jiexin Zhang, Songyuan Tang, Chao Chen, Hezhong Jiang, Hai Liao, Huawei Liu, Li Wang, Xin Chen

**Affiliations:** ^1^ Department of Laboratory Medicine, The Third People’s Hospital of Chengdu, Affiliated Hospital of Southwest Jiaotong University, Chengdu, Sichuan, China; ^2^ Faculty of Life Sciences and Engineering, Southwest Jiaotong University, Chengdu, Sichuan, China; ^3^ Department of Osteology, The 5th People’s Hospital of Jinan, Jinan, Shandong, China; ^4^ Sichuan Baicheng Chinese Medicine Technology Co., Chengdu, Sichuan, China

**Keywords:** hypobaric hypoxia, apnea syndrome, hippocampus, oxidative stress, bibliometric study

## Abstract

**Background:** The two main symptoms at high altitude, sleep abnormalities and cognitive impairments, interact with each other. These two dysfunctions are also closely related to systemic multisystem diseases, including cerebrovascular diseases, psychiatric disorders, and immune regulatory diseases.

**Purpose:** To systematically analyze and visualize research on sleep disturbances and cognitive impairment at high altitudes using a bibliometrics method, and to determine future research directions by analyzing research trends and the latest hotspots.

**Methods:** Publications from 1990 to 2022 on sleep disturbances and cognitive impairment at high altitudes were retrieved from the Web of Science. Using the R Bibliometrix software and Microsoft Excel, all data were examined statistically and qualitatively. For network visualization, the data were later exported into VOSviewer 1.6.17 and CiteSpace 6.1.R6.

**Results:** A total of 487 articles in this area were published from 1990 to 2022. In this period, there was an overall increase in the number of publications. The United States has shown considerable importance in this sector. Bloch Konrad E was the most prolific and valuable author. The most prolific journal was High Altitude Medicine & Biology, and it has been the first choice for publishing in this field in recent years. Analysis of keyword co-occurrences suggested that research interest in the clinical manifestations of sleep disturbances and cognitive impairment caused by altitude hypoxia was mainly focused on “acute mountain-sickness,” “insomnia,” “apnea syndrome,” “depression,” “anxiety,” “Cheyne-strokes respiration,” and “pulmonary hypertension.” The mechanisms of disease development related to “oxidative stress,” “inflammation,” “hippocampus,” “prefrontal cortex,” “neurodegeneration,” and “spatial memory” in the brain have been the focus of recent research. According to burst detection analysis, “mood” and “memory impairment,” as terms with high strength, are expected to remain hot topics in the coming years. High-altitude-induced pulmonary hypertension is also in the emerging stage of research, and the treatments will continue to receive attention in the future.

**Conclusion:** More attention is being focused on sleep disturbances and cognitive impairment at high altitudes. This work will serve as a useful reference for the clinical development of treatments for sleep disturbances and cognitive impairment induced by hypobaric hypoxia at high altitudes.

## 1 Introduction

High-altitude regions have extreme environments, including low atmospheric partial pressure, hypoxia, cold and dry conditions, and intense ultraviolet light, all of which have effects on human physiology and psychology, even endangering life and health (Zhang and Zhang, 2022). About 500 million people live in plateau areas worldwide, and more than 10 million of them have long lived on the Tibetan plateau, the world’s highest plateau at 2,200 m (Wu and Kayser, 2006). Every year, more than 100 million people travel the regions at high altitudes above 2,500 m (Netzer et al., 2013), and at least 10%–20% of them suffer from acute mountain sickness at these altitudes (Williamson et al., 2018). Physical and mental health challenges at high altitudes are becoming more prominent.

The geographical settings and climates of plateaus have a variety of impacts on people’s physiology and psychology, such as headaches, insomnia, and neurological dysfunctions, even developing into high-altitude cerebral edema and high-altitude pulmonary edema with high mortality (Imray et al., 2010; Netzer et al., 2013). Among all symptoms, insomnia is one of the most frequent complaints (Imray et al., 2010). Nearly half of people who first enter a plateau region suffer from sleep disturbances, which can be secondary to periodic breathing, shortness of breath, severe headaches, dizziness, and other causes (Imray et al., 2010). The altered sleep architecture is due to frequent arousals and reduced deep sleep caused by exposure to hypoxic environments (San et al., 2013). Altitude-induced periodic breathing is one of the manifestations of central sleep apnea, the main cause of which may be hypoxia-induced central inhibition that impairs respiratory sensory feedback mechanisms (Eckert et al., 2007). Cognitive impairment often coexists with sleep disorders. Respiratory impairment and reduced oxygen saturation lead to frontal gray matter atrophy after disrupting slow-wave sleep, further manifesting in reduced cognitive flexibility (Hermesdorf et al., 2021). Sleep disturbances affect brain function as well as information processing and storage by altering the density and morphology of dendritic spines in the central system (Raven et al., 2018). Intermittent hypoxia at high altitude induces an increase in reactive oxygen species (ROS), causing defects in synaptic plasticity in the hippocampal region and the death of hippocampal neurons, leading to memory and cognitive impairment (Knierim, 2015). Reduced total sleep time, sleep fragmentation, and oxidative stress are all strongly associated with decreased neurocognitive performance (San et al., 2013). These can affect people’s quality of life and can also be secondary to emotional distress. At high altitudes, the frequent occurrence of sleep disturbances can promote the development of cognitive impairment, perhaps even as part of a vicious cycle (San et al., 2013).

High-altitude environments induce sleep disturbances and cognitive impairment mainly through their hypobaric hypoxia characteristics. Hypoxia stimulates the peripheral chemoreceptors of the carotid body and affects respiratory rate and depth, causing sleep disorders such as central sleep apnea and obstructive sleep apnea (Wang H. et al., 2022). Cognitive impairment is due to the alteration of the physiological mechanism of the body by altitude hypoxia. In stress mechanisms, when the oxygen supply is insufficient, intracellular ROS, reactive nitrogen species (RNS), and other free radicals are produced in large quantities (Papandreou et al., 2006; Maiti et al., 2008b). This leads to cell damage and apoptosis, disrupting the balance of oxidative and antioxidant systems in the brain, and resulting in cognitive impairment (Maiti et al., 2008b). At the cellular level, long-term exposure to high-altitude hypoxia leads to high-altitude polycythemia (HAPC), in which red blood cells and blood viscosity are increased, resulting in cumulative changes in brain structure and function and reduced cognition (Li and Wang, 2022). HAPC can also lead to sleep disorders such as difficulty falling asleep and waking up easily at night, which in turn can lead to decreases in cognitive functions such as memory, concentration, and mental flexibility (Li and Wang, 2022). In terms of molecular mechanisms, the low-pressure hypoxic environment induces hypoxia-inducible factor (HIF), erythropoietin (EPO), and inflammatory factors that affect erythropoiesis and then lead to HAPC and affect cognitive function (Li and Wang, 2022). Among them, HIF-1α may lead to cerebral ischemia and hypoxia by promoting neuronal autophagy activation (Niu et al., 2018). In addition, hypobaric hypoxia-induced glutamate excitotoxicity and reduced acetylcholine levels may lead to cortical and hippocampal neuronal damage (Hota et al., 2010; Muthuraju et al., 2011).

Currently, few medicines are used clinically for hypoxic injury at high altitudes, and some of them, such as acetazolamide, sulfadiazine, dexamethasone, nimodipine, and benzodiazepines, can cause serious adverse effects, such as daytime drowsiness and significant withdrawal, therefore, while improving sleep and cognitive ability, their application is greatly limited (Wang et al., 2013). In addition to pharmacological treatment, plateau-induced hypoxic-ischemic brain injury can also be treated with hyperbaric oxygenation. Hyperbaric oxygen therapy can effectively improve gas exchange, increase tissue oxygen content and oxygen reserve, accelerate lactate clearance, reduce tissue damage from hypoxia, and decrease the incidence of sleep disturbances and cognitive impairment (Dou et al., 2016). However, hyperbaric oxygen therapy may lead to adverse effects such as tinnitus, barotrauma, oxygen toxicity, and decompression injury (Johnson-Arbor and Field, 2022). These adverse conditions have become pressing problems in clinical practice. The search for potential therapeutic targets and the exploration of relevant preventive and therapeutic approaches may provide new directions for the clinical treatment of sleep disorders and cognitive impairment caused by the high altitude.

Bibliometrics includes quantitative and qualitative analysis of bibliographic information by performing bibliometric analysis and building data matrices for co-citation, coupling, scientific collaboration analysis, and co-word analysis (Aria and Cuccurullo, 2017). Compared with other analytical methods, bibliometrics provides a more objective and reliable analysis (Aria and Cuccurullo, 2017). Scholars use bibliometric analysis to track the trends and characteristics of a field of research over a specific period of time (Aria and Cuccurullo, 2017). It mainly includes analysis of the sources and quantity of relevant literature, the study of the distribution and relevance of authors/countries/institutions, and prediction of the main development directions of the field of research in the future. Therefore, we conducted a bibliometric analysis and visualization of studies on sleep disorders and cognitive impairment at high altitudes published worldwide from 1990 to 2022 to discover the field’s research development and to explore future possible therapeutic targets and strategies.

## 2 Methods

### 2.1 Data collection

**FIGURE 1:**
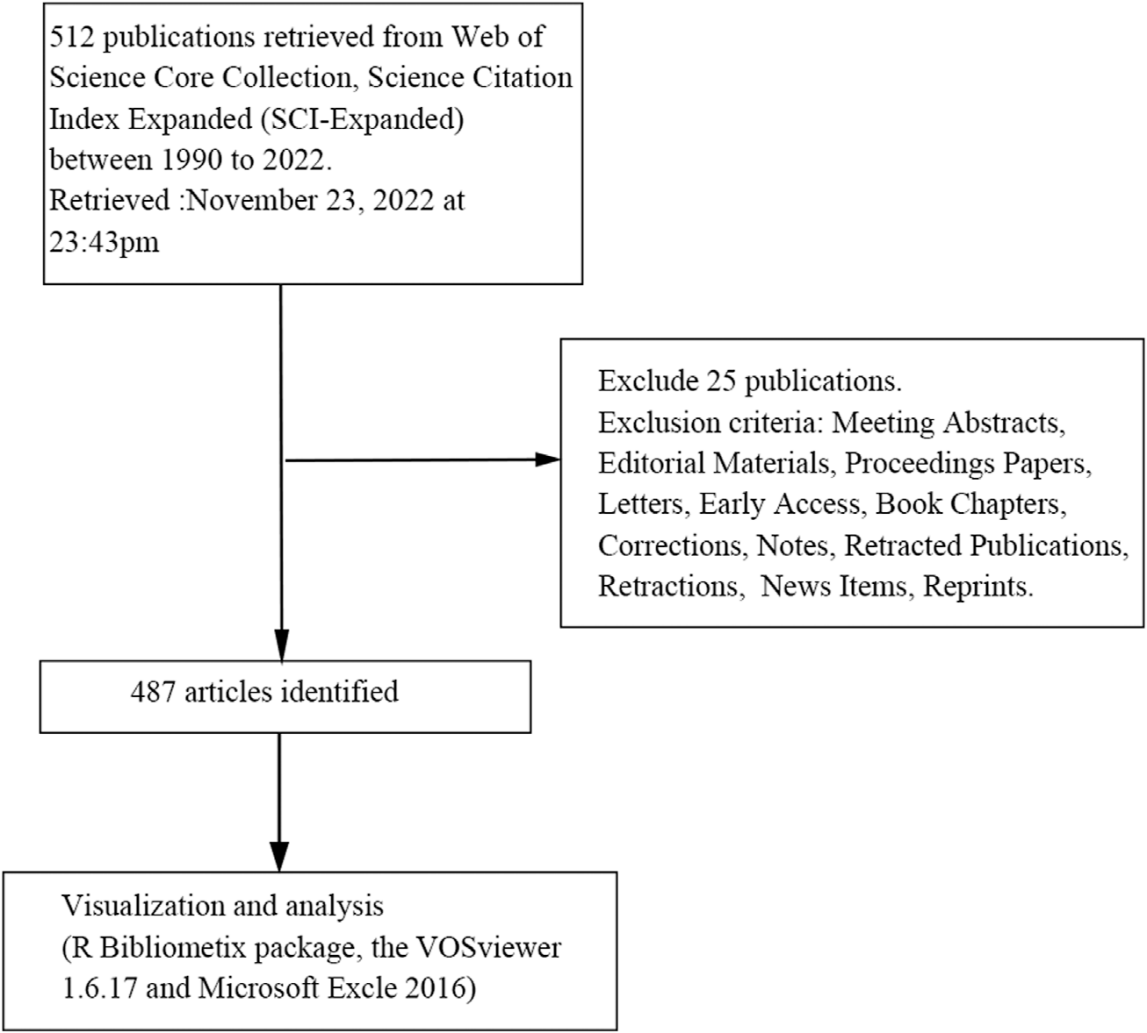
Flowchart of retrieval of publications on high-altitude-induced sleep disturbances and cognitive impairment from the Web of Science (WOS) Core Collection SCI-Expanded database, as well as article inclusion and exclusion criteria.

We retrieved relevant data from the Web of Science Core Collection (WOSCC) database on 23 November 2022, one of the most important databases of scholarly information in the world. The database has been around for more than half a century and includes the most important scholarly journals and international conferences, using a very rigorous selection mechanism. The WOSCC consists of six sub-collections, and we chose the Science Citation Index-Expanded, which is the most extensive and most complete core journal citation index database covering research from 1990 to the present, hoping to minimize the omission of relevant research from ancient times to the present (Yi et al., 2016). In order to avoid database update bias, we performed all the searches in 1 day. We employed a subject-words retrieval method and entered the following search terms: TS = (cognition disorder OR cognitive impairment OR cognitive dysfunction OR cognitive deficit OR dyssomnia OR sleep disorder OR insomnia OR sleep initiation and maintenance disorder) AND (high altitude OR hypobaric hypoxia OR altitude hypoxia). Using subject-word retrieval can obtain comprehensive and accurate results, and it includes retrieval from titles, abstracts, author keywords, and keywords plus. We restricted document types to articles and reviews, excluding all other types of publications (Meeting Abstracts, Editorial Materials, Proceedings Papers, Letters, Early Access, Book Chapters, Corrections, Notes, Retracted Publications, Retractions, Discussions, News Items, Corrections, Additions, Reprints), irrelevant articles, and repetitive or retracted publications. In the end, a total of 487 publications were selected. All data were downloaded in “BibTeX” and “Plain Text” file formats. The detailed data retrieval process and article inclusion criteria are summarized in Figure 1.

### 2.2 Analytical methods

We used four software tools to analyze and assess the authors, journals, institutions, countries, and keywords of the articles: VOSviewer 1.6.17, the Bibliometrix package, Microsoft Excel 2016, and CiteSpace 6.1.R6. Visualization was conducted using VOSviewer, a piece of software focusing on bibliometric networks and developed by the Centre for Science and Technology Studies at Leiden University. We used VOSviewer 1.6.17 (van Eck and Waltman, 2017) to create network maps of the data from WOS, to perform visual analysis, and to explore these maps, including the analysis of keyword co-occurrences and co-authorship networks of relevant countries/institutions. We utilized the Bibliometrix package in R 4.1.0 to convert and analyze the data of selected publications (Wallin, 2005), including general trends of publication, country/institution, and author distribution, and the analysis of documents. The trend of research in the field can be analyzed by the changes in the annual output of articles. To find the most influential and authoritative papers, we used the total local citations score (LCS) and the global citations score (GCS) (Salouti et al., 2020). We also used it to obtain co-occurrence networks, social structure relationships, and dynamic changes in the research areas around the world. Microsoft Excel was used to plot the overall number of publications on cognitive impairment at high altitudes to predict future trends. We put the “publication year subtract 1990” on the *X*-axis, the number of publications on the *Y*-axis, and drew the predict equation: y = 1E-97e^0.1122x^. CiteSpace 6.1.R6 is a citation visualization and analysis software that has been developed over time in the context of scientometrics and data visualization; it is a statistical analysis tool based on the Java environment and has been developed with the support of Prof. Mei-Chao Chen (Chen, 2004). The software uses the theory of co-citation analysis, and visual maps are drawn to analyze references and keywords in a specific field and to explore the development trends of that field. CiteSpace 6.1.R1 was used with the imported WOSCC data, and co-citation analysis as well as frontier analysis were performed.

## 3 Results

A total of 512 publications related to sleep disturbances and cognitive impairment at high altitudes were retrieved in the WOS Core Collection SCI-Expanded database. After article type filtering, only articles and reviews were retained, and in the end 487 articles met the requirements. These were exported in literature database (BibTeX) and plain text formats.

### 3.1 Evolution of scholarly interest in sleep disturbances and cognitive impairment at high altitudes over the period 1990–2022

**FIGURE 2:**
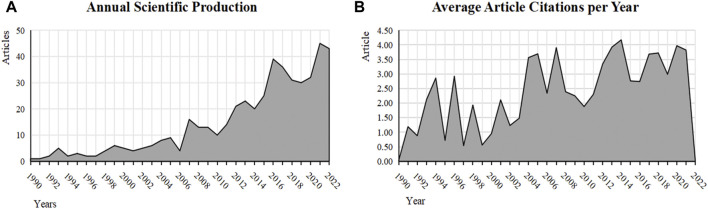
**(A)** Annual scientific production and **(B)** average article citations per year.

Collectively, 487 publications related to sleep disturbances and cognitive impairment at high altitudes were retrieved from the WOS Core Collection SCI-Expanded database, for the period 1990 to 2022. The number of scientific publications in this field showed an overall exponential increase with an annual growth rate of 12.47%. Since 2007, the average annual number of article citations in this area began to exceed ten articles (Figure 2A), and scholars have gradually increased their interest in high-altitude-induced sleep disturbances and cognitive impairment. In 10 of the 32 years from 1990 to 2022, there was an average annual number of article citations greater than three (Figure 2B). The highest average annual article citations were in 2013, 2014, and 2020.

### 3.2 Distribution by countries and affiliations

**FIGURE 3:**
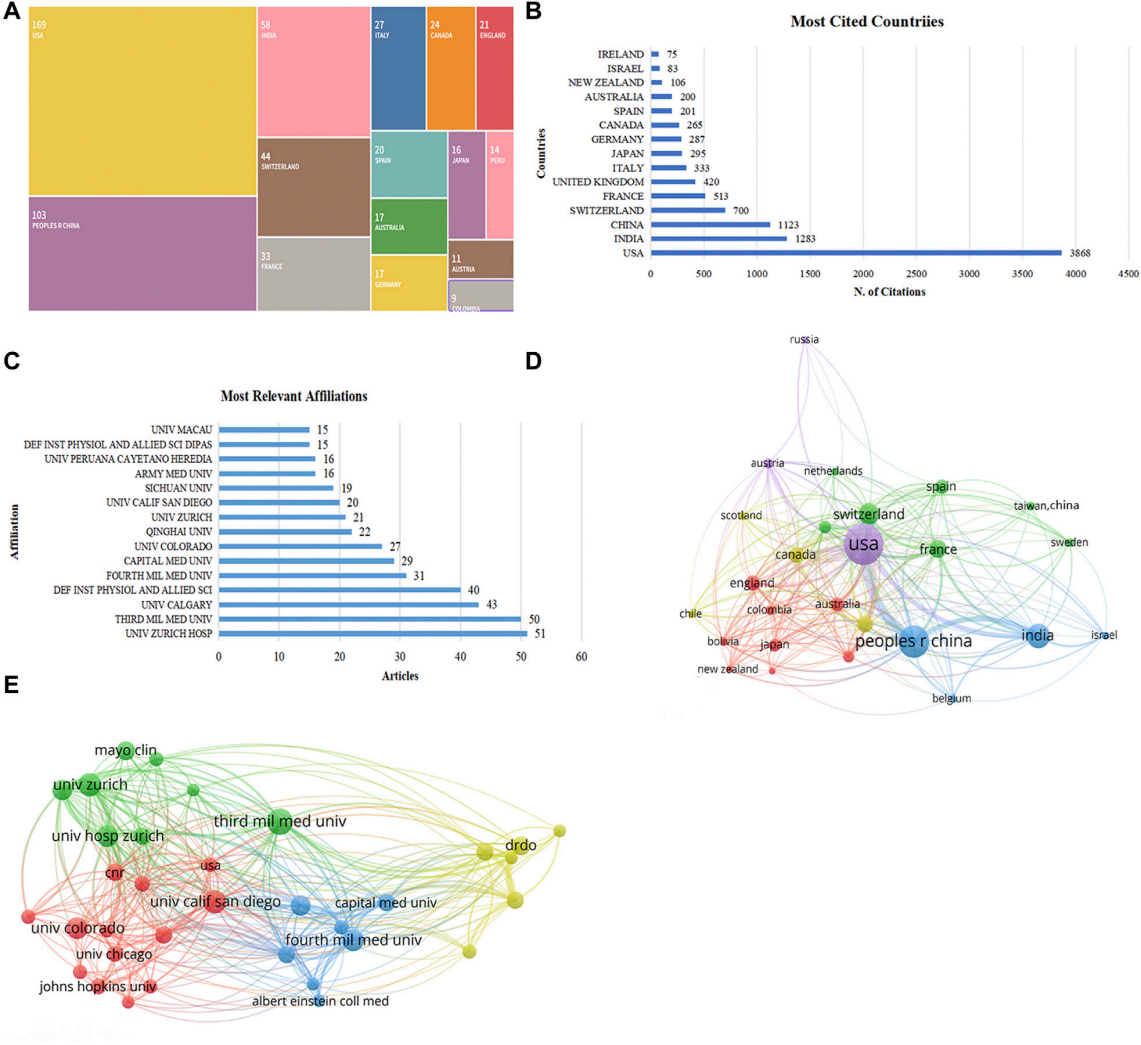
Country and affiliation contribution analysis. **(A)** The top 15 countries in terms of number of publications. **(B)** The top 15 countries cited the number of citations. **(C)** The top 15 most relevant affiliations and their output volume. **(D)** Co-authorship visualization analysis by countries. **(E)** Co-authorship visualization analysis by affiliations (Larger circles represent more output from a country or affiliation, and more lines represent closer interconnections).

Over the past 32 years, total of 53 countries have contributed to research on sleep disturbances and cognitive impairment at high altitudes. The issue of altitude-induced sleep disturbances and cognitive impairment has attracted the attention of researchers globally, with Asia, North America, and Europe being the regions of the most extensive study. The United States was the most prominent contributor in this area, leading in both scientific publications and citations, with 169 publications and 3,868 citations (Figures 3A, B), followed by China (103 publications and 1,123 citations), India (58 and 1,283), Switzerland (40 and 700), France (33 and 513), and the Italy (27 and 420). Although India published far fewer articles than China, it was second only to the United States in terms of citations, indicating that India has been recognized worldwide for its research on plateau-induced sleep disorders and cognitive impairment.

A total of 892 institutions had conducted research on sleep disturbances and cognitive impairment at high altitudes, predominantly universities and supplemented by research institutes and hospitals. We list the top 15 affiliations in Figure 3C based on the number of publications. Thirty-one institutions had published more than ten studies in this field, with the top-ranked being the University Hospital Zurich with 51 publications, followed by the Third Military Medical University (50 publications), the University of Calgary (43), the Defence Institute of Physiology and Allied Sciences (40), and the Fourth Military Medical University (31).

Beyond that, we performed a visual analysis of co-authorship for countries and affiliations that published more than five studies, with 26 countries and 34 institutions meeting the threshold (Figures 3D, E). The top five countries with the highest total link strength were the United States (total link strength = 847), China (549), Switzerland (356), India (316), and France (224). The top five affiliations with the highest total link strength were the University Hospital Zurich (174), the University of Zurich (99), the Defence Institute of Physiology and Allied Sciences (71), Sichuan University (66), and the Fourth Military Medical University (63). A higher total link strength indicates a higher intensity of collaboration and association between countries/institutions.

### 3.3 Contribution by journals and authors

**FIGURE 4:**
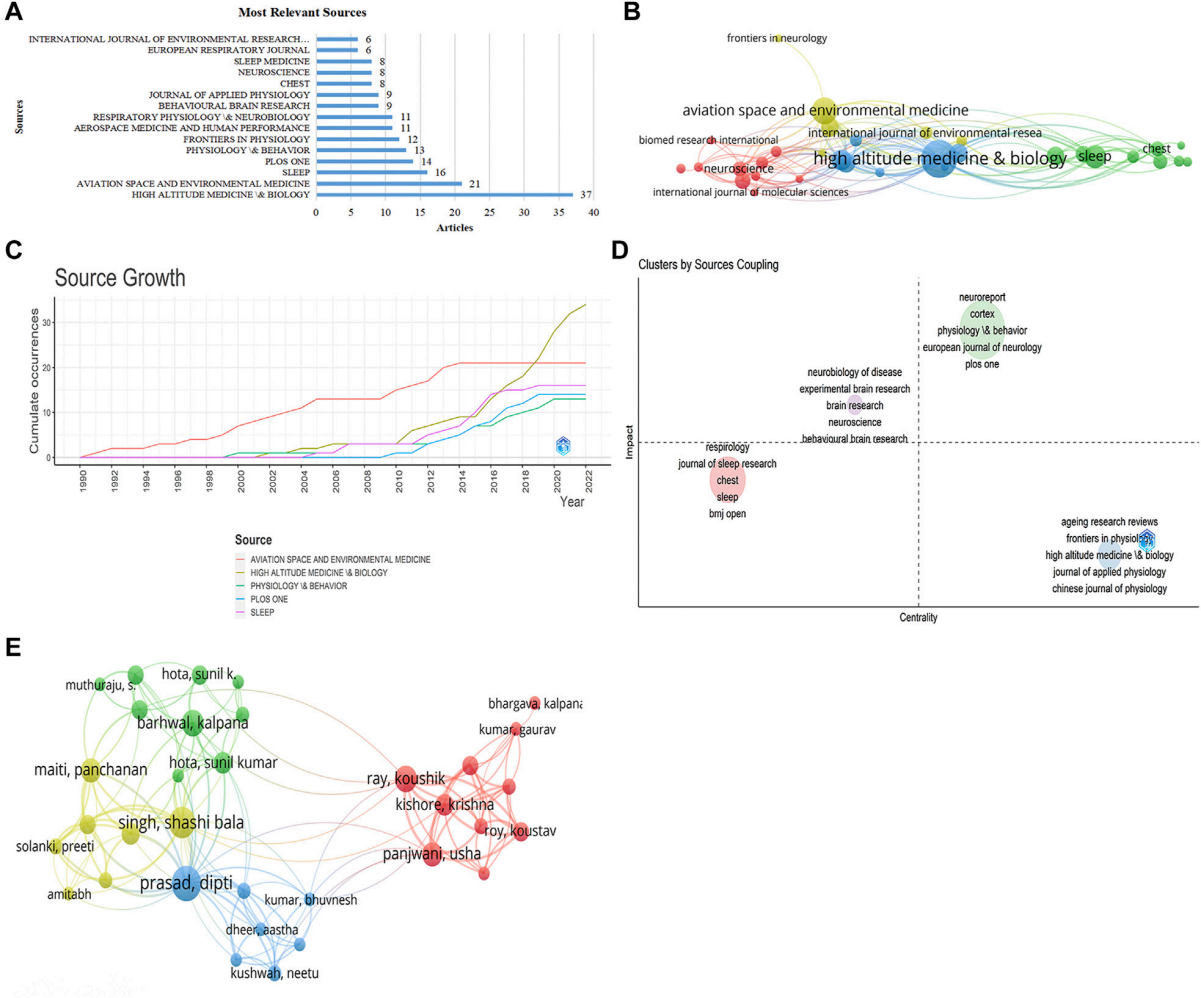
Journal and author contribution analysis. **(A)** The top 15 most relevant journals and their output volume. **(B)** Citation analysis of relevant journals (the larger the circle, the more references to them). **(C)** Publication trends for the top five journals. **(D)** Dimension analysis of journals (horizontal axis: centrality, vertical axis: density). **(E)** Co-authorship of some related authors (larger circles represent higher output by an author, and more lines represent closer interconnections).

On this subject, 231 journals had published relevant research, among which there were 20 journals that had published more than five studies. There were 37 studies published in Aerial Medicine and Biology, accounting for 16.0% of all publications. This was followed by Aerospace & Environmental Medicine (21 publications, 9.09%), Sleep (16, 6.93%), PLOS One (14, 6.06%), and Physiology & Behavior (13, 5.63%) (Figure 4A). Although Aerospace and Environmental Medicine did not rank first in terms of publication volume, it was the most cited by peers (655 citations), indicating that publications in this journal had greater academic impact and higher professional recognition. It was followed by Sleep (546 citations), PLOS One (439), Chest (400), and the Journal of Applied Physiology (380) (Table 1). For visual clarity, we set the threshold that only journals that had published at least three relevant studies would be presented in the co-authorship analysis network, and 33 journals met that threshold (Figure 4B). The top five journals with the highest total link strengths were High Altitude Medicine & Biology (total link strength = 109), Aerospace Medicine and Human Performance (90), Physiology and Behavior (79), PLOS One (58), and Sleep (43). The source dynamic growth line graph shows that Aerospace Medicine and Human Performance was the biggest contributor to the field until 2019, when the volume of papers in High Altitude Medicine & Biology started to lead (Figure 4C). We present detailed citation information for the top 20 high-producing journals that published relevant research in Table 1. Furthermore, we created a strategic map of the macro presentation by coupling the analysis of relevant journals with a dimensional analysis of impact and density (Figure 4D). Journals located in the middle green circle represent the most influential journals in the field, with high-quality publications and broad impact; these include Neuroreport, Cortex, and Physiology & Behavior. Journals in the purple area, namely, Neurobiology of Disease, Experimental Brain Research, and Brain Research, have emerged in the field in recent years and have greater potential for future growth. The journals that fall within the blue circles are well-developed, but some appear to have less impact in the field, including the Review of Aging Research, Frontiers in Physiology, and High Altitude Medicine & Biology. Additionally, Respirology, the Journal of Sleep Research, and Chest are in the red circle in the third quadrant, indicating that publications in these journals are marginal and may have no significant impact.

**TABLE 1:** The top 20 most productive journals for publications on high-altitude induced disturbances and cognitive impairment.

Sources	Publications	Citation	Total link strength
High Altitude Medicine & Biology	37	344	109
Aviation Space and Environmental Medicine	21	655	90
Sleep	16	546	43
PLos One	14	439	58
Physiology & Behavior	13	294	79
Frontiers in Physiology	12	130	41
Aerospace Medicine and Human Performance	11	76	31
Respiratory Physiology & Neurobiology	11	324	18
Behavioural Brain Research	9	199	41
Journal of Applied Physiology	9	380	36
Chest	8	400	23
Neuroscience	8	184	37
Sleep Medicine	8	65	15
European Respiratory Journal	6	337	32
International Journal of Environmental Research and Public Health	6	25	10
Neurochemical Research	6	63	18
Wilderness & Environmental Medicine	6	93	29
Frontiers in Psychiatry	5	14	19
Scientific Reports	5	50	8
Sleep and Breathing	5	86	8

In the 487 included publications, a total of 2,122 authors were involved in the research on plateau-induced sleep disturbances and cognitive impairment. We selected 45 authors with more than five publications each for co-authorship network visualization (Figure 4E). Bloch Konrad E was the most productive author with 13 publications, followed by Prasad Dipti (12 publications), Singh Shashi Bala (10), and Luo Wenjing (8). Bloch Conrad E’s research was widely recognized and accepted by scholars worldwide, and his studies had been cited up to 338 times, followed by Maiti Panchanan (329 citations), Prasad Dipti (271), Singh Shashi Bala (232), and Muthuraju Sangu (214). Although Muthuraju Sangu published only five studies in this field, his total citations were ranked in the top five. On the other hand, according to the co-authorship network, the top five authors with the highest total link strength were Prasad Dipti (total link strength = 60), Singh Shashi Bala (49), Huang Lan (45), Yu Jie (45), and Zhang Jihang (45). There was close collaboration between them and other scholars working on plateau-induced sleep disturbances and cognitive impairment.

### 3.4 Analysis of reference citations and co-citation

**FIGURE 5:**
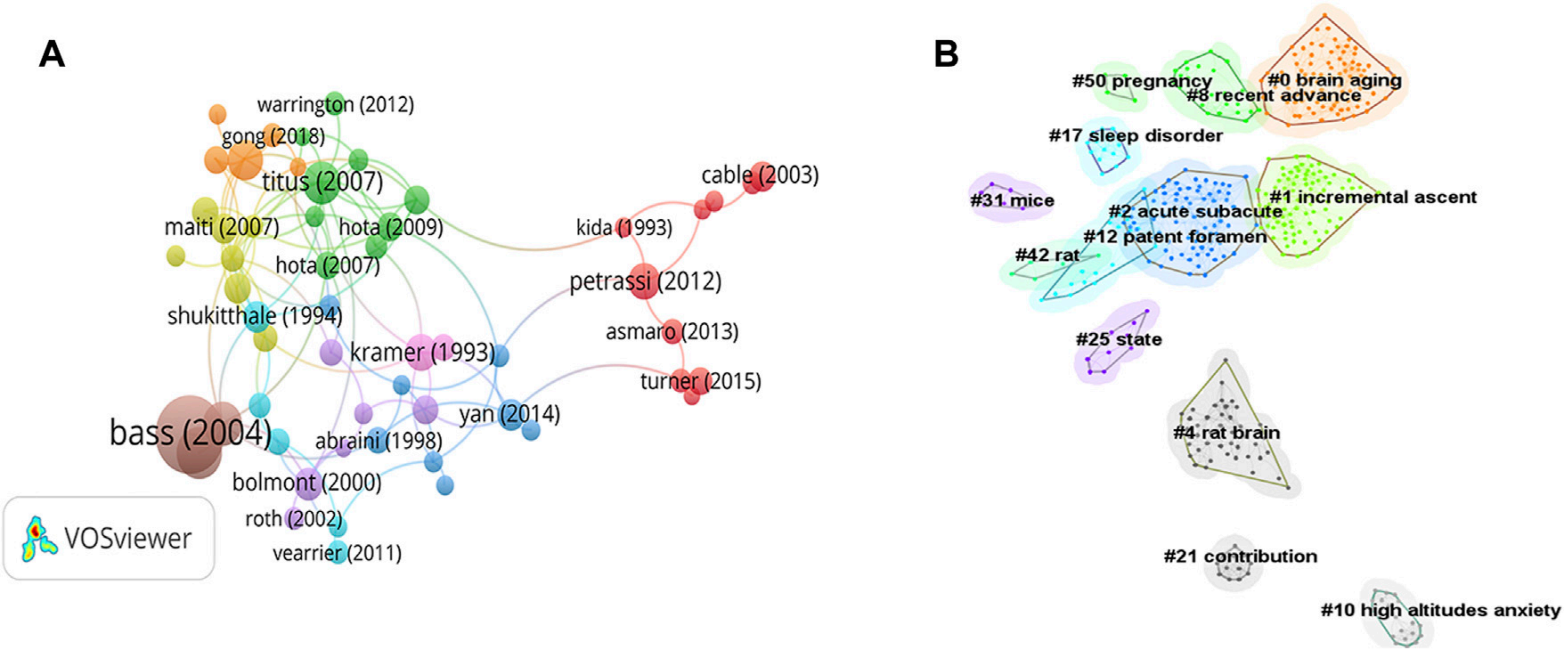
Analysis of reference citations and co-citations. **(A)** Citation analysis of relevant documents. **(B)** Knowledge map of co-cited reference analysis. (Different circles represent different clusters, each node represents different publications).

The inclusion/exclusion criteria yielded 487 publications for subsequent analysis. The total number of citations reached 10,044. According to the GSC ranking, the most cited publication had 301 citations. Twelve publications were cited more than 100 times, and 53 publications were cited more than 50 times (Figure 5A; Table 2). Moreover, we listed information for the top 20 publications according to LCS ranking (Table 3). Maiti led a team that explored the behavioral, biochemical, and morphological changes after hypobaric hypoxia of different durations and they proposed that memory impairment at high altitudes is due to apoptosis in the hippocampus, cortex, and striatum (Maiti et al., 2008b). The findings were widely recognized and cited by scholars around the world and became the most locally cited study in the field (32 LCS, 101 GCS); this was followed by “Cognitive function at high altitude” by Kramer et al. in 1993 (31 LCS, 86 GCS) (Kramer et al., 1993), and then “Hypobaric hypoxia-induced dendritic atrophy of hippocampal neurons is associated with cognitive impairment in adult rats” by Titus et al. in 2007 in Neuroscience (31 LCS, 113 GCS) (Titus et al., 2007). In 2015, Clare et al. conducted a randomized controlled trial to present the first experimental model for inducing global hypoxia (Turner et al., 2015). By simulating the metabolic crisis that occurs with sudden exposure to a reduced oxygen supply, it was clinically demonstrated that the frontal cortex and hippocampus are abnormally vulnerable when oxygen delivery is compromised. The model proposed in that trial induced a decline in neuropsychological performance comparable to the cognitive decline induced by plateau exposure and mild traumatic brain injury, providing a new approach for simulating the metabolic–cognitive crises in the early stages of exposure to the plateau, respiratory device failure, or mild traumatic brain injury.

**TABLE 2:** The top-cited publications with more than 50 citations.

Paper	Total citations	DOI
Bass JL, 2004, Pediatrics	301	10.1542/peds.2004-0227
Eckert DJ, 2007, Chest	264	10.1378/chest.06.2287
Navarrete-Opazo A, 2014, Am J Physiol-Regul Integr Comp Physiol	222	10.1152/ajpregu.00208.2014
Thomas RJ, 2005, Sleep	168	10.1093/sleep/28.9.1151
Thomas RJ, 2005, J Appl Physiol	154	10.1152/japplphysiol.01225.2004
Bahrke MS, 1993, Sports Med	116	10.2165/00007256-199316020-00003
Randerath W, 2017, Eur Resp J	116	10.1183/13993003.00959-2016
Titus Adj, 2007, Neuroscience	113	10.1016/j.neuroscience.2006.11.037
Javaheri S, 2013, Compr Physiol	104	10.1002/cphy.c110057
Dykiert D, 2012, PLos One	104	10.1371/journal.pone.0045759
Labanowski M, 1996, Neurology	102	10.1212/WNL.47.5.1173
Maiti P, 2008, J Chem Neuroanat	101	10.1016/j.jchemneu.2008.07.003
Kayser B, 2013, Obes Rev	99	10.1111/obr.12034
Dematteis M, 2009, Ilar J	92	10.1093/ilar.50.3.262
Nussbaumer-Ochsner Y, 2012, Sleep	91	10.5665/sleep.1708
Krasney JA, 1994, Med Sci Sports Exerc	90	10.1249/00005768-199402000-00010
Brugniaux JV, 2007, Respir Physiol Neuro	90	10.1016/j.resp.2007.04.008
Petrassi FA, 2012, Aviat Space Environ Med	87	10.3357/ASEM.3315.2012
Kramer AF, 1993, Hum Factors	86	10.1177/001872089303500208
Imagawa S, 2001, Blood	79	10.1182/blood.V98.4.1255
Kumar K, 2018, Biomed Pharmacother	78	10.1016/j.biopha.2017.12.053
Bailey DM, 2001, Aviat Space Environ Med	75	NA
Luks AM, 2007, Eur Resp J	74	10.1183/09031936.00052606
Bolmont B, 2000, Physiol Behav	74	10.1016/S0031-9384(00)00362-0
Mishra CB, 2020, Med Res Rev	74	10.1002/med.21713
Shukitthale B, 1994, Behav Neural Biol	70	10.1016/S0163-1047(05)80023-8
Yan X, 2014, High Alt Med Biol	70	10.1089/ham.2014.1009
Bradford A, 2005, Respir Physiol Neuro	68	10.1016/j.resp.2005.04.001
Fischer R, 2004, Eur Resp J	67	10.1183/09031936.03.00113102
Muthuraju S, 2009, Behav Brain Res	64	10.1016/j.bbr.2009.03.026
Cable GG, 2003, Aviat Space Environ Med	63	NA
Maiti P, 2007, Brain Res	62	10.1016/j.brainres.2007.06.106
Kadar T, 1998, J Neural Transm	62	10.1007/s007020050107
Hota SK, 2008, Neurobiol Learn Mem	359	10.1016/j.nlm.2008.01.003
Yan X, 2011, Exp Brain Res	59	10.1007/s00221-010-2494-x
Chen HC, 2013, Sleep	59	10.5665/sleep.2884
Hota SK, 2009, Neurobiol Dis	58	10.1016/j.nbd.2008.12.006
Chiang AA, 2006, Chin J Physiol	58	NA
Ando S, 2013, PLos One	58	10.1371/journal.pone.0063630
Liu P, 2015, Restor Neurol Neurosci	57	10.3233/RNN-140446
Turner CE, 2015, Physiol Behav	57	10.1016/j.physbeh.2015.02.006
Hota SK, 2007, Neurochem Int	56	10.1016/j.neuint.2007.04.003
Said SI, 2006, Am J Physiol-Lung Cell Mol Physiol	56	10.1152/ajplung.00546.2005
Rodway GW, 2003, Heart Lung	54	10.1016/j.hrtlng.2003.08.002
Smolensky MH, 2015, Sleep Med Rev	54	10.1016/j.smrv.2014.07.001
Lombardi C, 2013, J Sleep Res	53	10.1111/jsr.12012
Arya A, 2016, Int J Nanomed	53	10.2147/IJN.S102096
Maiti P, 2008, Behav Brain Res	53	10.1016/j.bbr.2008.01.007
Wang J, 2013, Neurotoxicol Teratol	52	10.1016/j.ntt.2012.12.003
Burgess KR, 2004, Respirology	52	10.1111/j.1440-1843.2004.00625.x
Abraini JH, 1998, Pflugers Arch	52	10.1007/s004240050671
Paola MD, 2008, Eur J Neurol	50	10.1111/j.1468-1331.2008.02243.x

**TABLE 3:** Information about the top 20 most locally cited publications.

Document	DOI	Year	Local citations	Global citations	LC/GC ratio (%)
Maiti P, 2008, J Chem Neuroanat	10.1016/j.jchemneu.2008.07.003	2008	32	101	31.68
Kramer AF, 1993, Hum Factors	10.1177/001872089303500208	1993	31	86	36.05
Titus Adj, 2007, Neuroscience	10.1016/j.neuroscience.2006.11.037	2007	31	113	27.43
Shukitthale B, 1994, Behav Neural Biol	10.1016/S0163-1047(05)80023-8	1994	29	70	41.43
Bahrke MS, 1993, Sports Med	10.2165/00007256-199316020-00003	1993	26	116	22.41
Turner CE, 2015, Physiol Behav	10.1016/j.physbeh.2015.02.006	2015	24	57	42.11
Maiti P, 2008, Behav Brain Res	10.1016/j.bbr.2008.01.007	2008	22	53	41.51
Hota SK, 2008, J Neurosci Res	10.1002/jnr.21554	2008	21	47	44.68
Nussbaumer-Ochsner Y, 2012, Sleep	10.5665/sleep.1708	2012	17	91	18.68
Bolmont B, 2000, Physiol Behav	10.1016/S0031-9384(00)00362-0	2000	16	74	21.62
Pagani M, 1998, Cortex	10.1016/S0010-9452(08)70751-2	1998	15	27	55.56
Maiti P, 2007, Brain Res	10.1016/j.brainres.2007.06.106	2007	14	62	22.58
Gao YX, 2015, Eur J Neurol	10.1111/ene.12507	2015	14	28	50.00
Jayalakshmi K, 2007, Physiol Behav	10.1016/j.physbeh.2007.05.051	2007	13	40	32.50
Hota SK, 2007, Neurochem Int	10.1016/j.neuint.2007.04.003	2007	12	56	21.43
Muthuraju S, 2009, Behav Brain Res	10.1016/j.bbr.2009.03.026	2009	12	64	18.75
Burgess KR, 2004, Respirology	10.1111/j.1440-1843.2004.00625.x	2004	11	52	21.15
Bloch KE, 2015, J Appl Physiol	10.1152/japplphysiol.00448.2015	2015	11	39	28.21
Van Der Post J, 2002, J Psychopharmacol	10.1177/026988110201600408	2002	10	34	29.41
Heinrich EC, 2019, PLos One	10.1371/journal.pone.0217089	2019	10	20	50.00

Co-citation analysis is the study of the relationship between documents. If two documents are cited by other documents at the same time, the two articles are said to have a co-citation relationship. It is generally believed that the topics of co-cited literature will be similar, so co-citation intensity can detect the relevance of the co-cited literature in terms of content. A total of 487 publications were analyzed using CiteSpace, and 13 clusters were extracted using the clustering algorithm. The LLR (log-likelihood ratio) algorithm was used to label the clusters, and the larger the silhouette value, the greater was the similarity of the clusters. The silhouette value for each cluster as shown in Table 4, was greater than 0.9, indicating that the clustering results were convincing. The average year of cluster 0 was 2018, showing that the research of this cluster was relatively fresh and on topics that have attracted attention only in recent years. The rest of the clustering years were mainly concentrated around 2010. We performed a co-citation analysis of cited references (Figure 5B). Different circles represent different clusters, each node represents a different publication, and nodes are connected to one another by lines that represent the network relationships among the publications. Figure 5B shows the complex integration among the 13 clusters, some of which are interdependent but independent of each other. The three largest clusters among the 13 clusters mentioned “hippocampal damage,” “neurovascular coupling,” and “psychomotor vigilance,” which are all related to basic research on sleep and cognitive impairment due to hypoxia in highlands.

**TABLE 4:** Summary of 13 clusters.

Cluster ID	Size	Silhouette	Mean(year)	Top terms (log-likelihood ratio)
0	100	0.946	2018	Brain aging (57.29, 1.0E-4); hippocampal damage (57.29, 1.0E-4); traumatic brain injury (54.54, 1.0E-4); potent antioxidant compound (54.54, 1.0E-4); hemorheological properties (51.79, 1.0E-4)
1	91	0.912	2015	Incremental ascent (82.05, 1.0E-4); neurovascular coupling (77.15, 1.0E-4); cognitive function (60.63, 1.0E-4); neurovascular function (43.11, 1.0E-4); cognitive activity (41.5, 1.0E-4)
2	82	0.913	2011	Acute subacute (92.48, 1.0E-4); psychomotor vigilance (47.29, 1.0E-4); acute altitude exposure (45.61, 1.0E-4); sleep quality change (45.61, 1.0E-4); cognitive functioning (44.18, 1.0E-4)
4	44	0.967	2007	Rat brain (97.11, 1.0E-4); hypobaric hypoxia (70.26, 1.0E-4); hippocampal pyramidal neuron (48.04, 1.0E-4); oxidative stress (48.04, 1.0E-4); derived peptide (48.04, 1.0E-4)
8	24	0.971	2013	Recent advance (50.14, 1.0E-4); induced hypobaric hypoxia (41.65, 1.0E-4); ginkgo biloba (33.22, 1.0E-4); molecular correlate (33.22, 1.0E-4); hypobaric hypoxia (26.73, 1.0E-4)
10	20	1	1998	High altitudes anxiety (14.86, 0.001); panic attack (14.86, 0.001); high altitude (0.46, 0.5); cognitive function (0.16, 1.0); acute mountain sickness (0.09, 1.0)
12	19	0.946	2013	Patent foramen (28.78, 1.0E-4); cardiovascular risk factor (28.78, 1.0E-4); susceptible patient (19.09, 1.0E-4); ventricular dysfunction (19.09, 1.0E-4); grover conference (19.09, 1.0E-4)
17	15	0.991	2013	Sleep disorder (22.95, 1.0E-4); 450-m altitude (22.95, 1.0E-4); comparison (22.95, 1.0E-4); sleep (11.35, 0.001); breathing (11.35, 0.001)
21	12	0.999	2006	Contribution (12.63, 0.001); understanding (12.63, 0.001); human disease (12.63, 0.001); cardiovascular consequence (12.63, 0.001); sleep-disordered breathing (5.07, 0.05)
25	9	0.99	2009	State (12.63, 0.001); science (12.63, 0.001); review (12.63, 0.001); hypoxic hypoxia (12.63, 0.001); moderate altitude (9.86, 0.005)
31	7	0.997	2010	Mice (13.81, 0.001); male fertility (13.81, 0.001); chronic intermittent hypoxia (13.81, 0.001); high altitude (0.69, 0.5); cognitive function (0.24, 1.0)
42	5	0.988	2013	Rat (20.71, 1.0E-4); reoxygenation-induced cognitive deficit (20.71, 1.0E-4); induction (20.71, 1.0E-4); cerebral cortex microglia (20.71, 1.0E-4); inducible nitric oxide synthase (20.71, 1.0E-4)
50	4	1	2015	Pregnancy (13.81, 0.001); management (13.81, 0.001); obstructive sleep apnea (13.81, 0.001); high altitude (0.69, 0.5); cognitive function (0.24, 1.0)

### 3.5 Research focus

**FIGURE 6:**
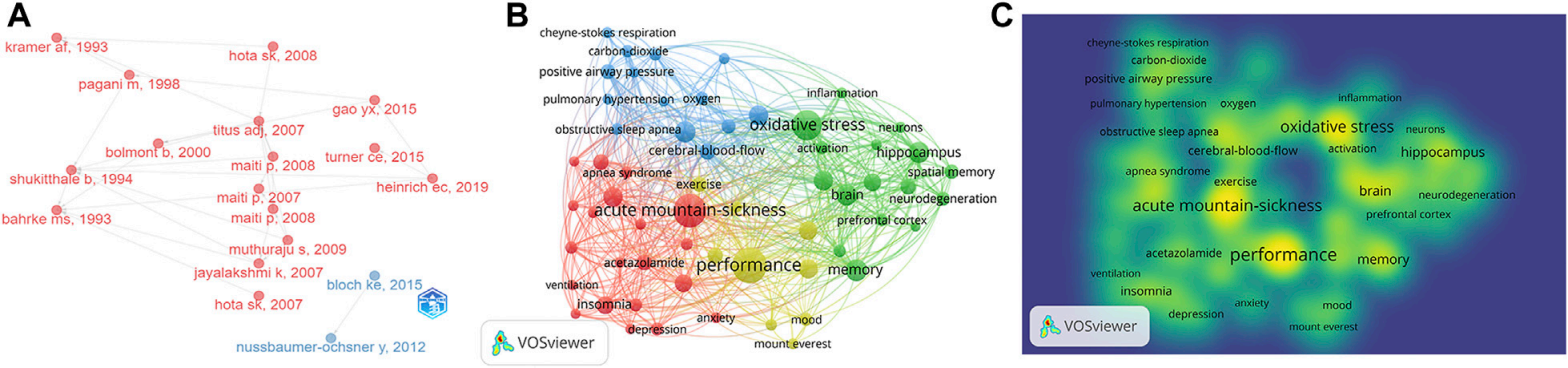
Research hotspot analysis. **(A)** Relevant literature history evolution analysis. **(B)** Visualization of literature keywords (the larger the circle, the higher the frequency of the word; the thicker the line, the closer the relationship between two words). **(C)** Density analysis of article keywords (yellow indicates high frequency).

Up to now, many studies have been conducted on sleep disturbances and cognitive impairment at high altitudes, and there have been some breakthroughs in this area. We performed a literature history evolution analysis to illustrate the history of the development of research on altitude-hypoxia-induced sleep disturbances and cognitive impairment (Figure 6A). In 1993, Kramer et al. first assessed the effects of high altitude on human cognitive performance (Kramer et al., 1993). Then, Shukitt proposed impaired spatial memory in an altitude-dependent manner (Shukitt-Hale et al., 1994). Since 2007, scholars have been extensively engaged in exploring the brain mechanisms underlying altitude-induced sleep disturbances and cognitive impairment. Titus and Maiti proposed in 2007 that cognitive impairment under high-altitude exposure may be associated with the CA region of the hippocampus (Maiti et al., 2007; Titus et al., 2007), and their findings were broadly recognized and accepted in the field. Subsequently, Maiti led a team that continued exploring this field and in 2008 they proposed (Maiti et al., 2008b) that increased levels of oxidative stress, reduced levels of antioxidants, and neurodegeneration in the brain were all associated with memory impairment due to hypoxia at high altitudes. In 2019, Heinrich suggested that adaptive servo-ventilation and supplemental oxygen therapy could remit altitude-induced sleep disturbances and improve cognitive function and mood (Heinrich et al., 2019).

To better understand the research topics, we performed a cluster visualization analysis of all keywords through the VOS viewer. We examined 17,670 keywords from the 487 publications (Table 5). To obtain better results in the cluster analysis, we set the minimum number of occurrences of the keywords to ten, and in the end, 83 keywords met that threshold. “Performance” had the highest number of occurrences (74) and total link strength (338), followed by “acute mountain sickness” (66 occurrences and total link strength = 301), “oxidative stress” (55 and 231), “memory” (34 and 166), and “hippocampus” (31 and 154). The co-occurrence network divided the keywords into three themes: 1) The specific manifestations of sleep disturbances at high altitudes, such as insomnia, obstructive sleep apnea, intermittent hypoxia, and sleep-disordered breathing. 2) Manifestations of cognitive impairment induced by altitude hypoxia, such as neurodegeneration, Alzheimer’s-disease, memory impairment, and anxiety. 3) The pathological mechanisms of hypobaric hypoxia-induced occurrence, such as oxidative stress and inflammation (Figures 6B, C).

**TABLE 5:** The 20 top-occurrence keywords from VOSviewer.

Keywords	Occurrence	Total link strength	Keywords	Occurrence	Total link strength
Performance	74	338	Cognitive performance	25	91
Acute mountain-sickness	66	301	Expression	24	114
Oxidative stress	55	231	Acclimatization	23	97
Memory	34	166	Insomnia	23	94
Obstructive sleep-apnea	32	119	Cerebral-blood-flow	21	93
Brain	31	143	Memory impairment	20	93
Apnea	29	119	Cognitive performance	20	90
Alzheimers-disease	28	117	Acetazolamide	19	93
Intermittent hypoxia	28	117	Sleep-disordered breathing	19	92
Working-memory	26	118	Exercise	18	83

### 3.6 Research frontiers analysis

**FIGURE 7:**
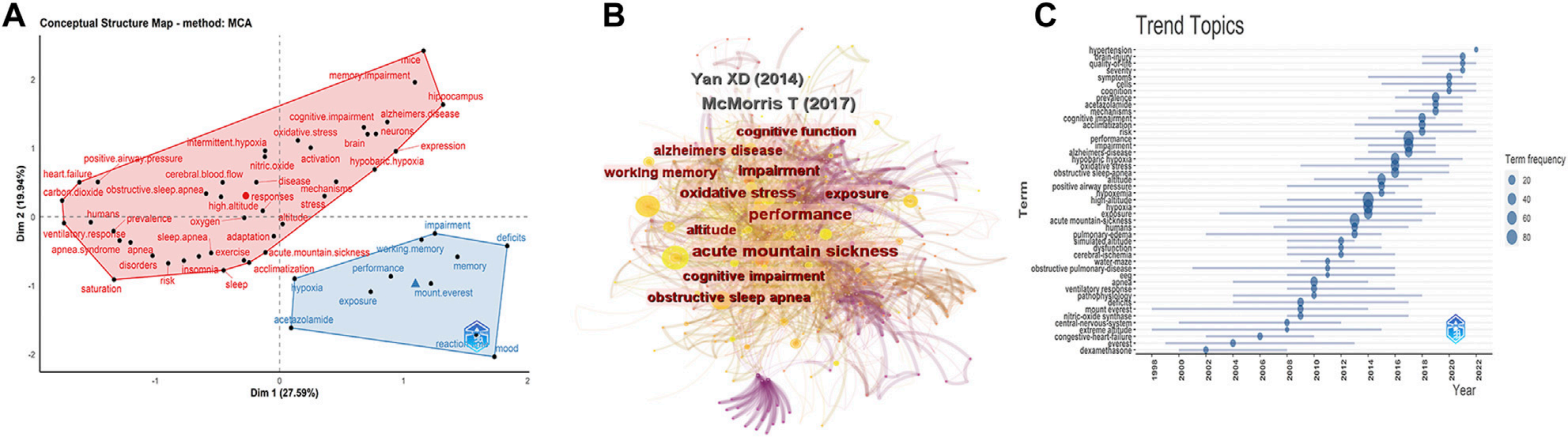
Research frontiers analysis. **(A)** Conceptual structure map by MCA method (classification of research areas into different categories based on keyword analysis). **(B)** Map of burst detection of emerging terms and references (light yellow representing keywords and purple representing references). **(C)** Trend topics prediction.

We employed a multiple correspondence analysis (MCA), a data simplification technique for creating a two-dimensional graphical map of scientific conceptual structures, to create conceptual structures for the top 50 keywords. In addition, the similarity of the analyzed words was taken into account. The closer and more similar the distribution of related words was, the more representative they were. In Figure 7A, the red areas show the topics of current focus in the field, while blue areas represent emerging, challenging, or unexplored topics. The left part of the red area included the vocabulary of sleep disorders, including apnea syndrome, insomnia, obstructive sleep apnea, acute mountain sickness, etc. The right part of the red area included cognitive aspects such as memory impairment, Alzheimer’s disease, neurons, etc. The blue terms, such as reaction time, working memory, and acetazolamide, were those which showed more potential for exploration in the future.

Burst detection captures values with large variability in the short term and we used it to detect the decline or rise of interest in particular subject terms. Figure 7B shows the burst detection graph for novel terms and citations. There were 822 nodes and 4,170 links (density = 0.0124), with light yellow representing keywords, proportional to their intermediate centrality, and purple representing references. The papers of Yan (2014) and McMorris et al. (2017) had high centrality values. As shown in Table 6, a total of 15 keywords were extracted. Among these, “apnea” had an explosive intensity of 3.47 and its influence had lasted for 17 years, starting from 1996 and lasting until 2013. “Reaction time” had the strongest explosive intensity of 5.34. “Memory impairment” and “mood” were terms that have continued to emerge in recent years and are likely to continue to emerge. Table 6 shows the 20 references with the strongest citation burst in the WOSCC database from 1990 to 2022. The citation explosion was mainly concentrated between 2007 and 2022. The paper of Yan X. et al. had the highest strength in citation burst (strength = 8.71), indicating that the article received strong attention from scholars in the field (Yan, 2014). This paper not only described the cognitive impairments associated with exposure to high altitude but also mentioned potential adaptive mechanisms in high-altitude residents that may be associated with some irreversible neuronal damage. There were three papers with high citation explosion intensity in 2019–2022, namely, Roach et al. (2018), Chen et al. (2017), and Pun et al. (2018), which were the cutting-edge research in this field in recent years. Among them, Chen X. et al. explored cognitive impairment, including working memory and psychomotor function impairment after long-term exposure to high altitude, while Pun M. et al. studied cognitive impairment caused by short-term exposure to a high-altitude zone, including selective and persistent attention impairment.

**TABLE 6:** The top 15 keywords with the strongest bursts from 1990 to 2022 according to CiteSpace.

Keywords	Strength	Begin	End	1990–2022
Exposure	2.87	1991	2003	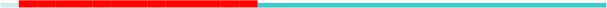
Deficit	3.48	1994	2009	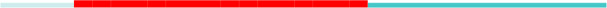
Apnea	3.47	1996	2013	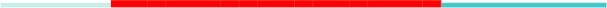
Acute mountain sickness	2.89	1999	2004	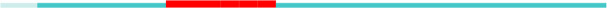
Heart failure	3.97	2006	2016	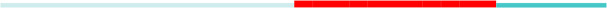
Simulated altitude	3.88	2007	2013	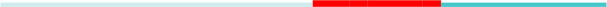
Insomnia	3.74	2012	2014	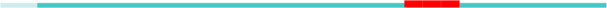
Positive airway pressure	3.62	2014	2018	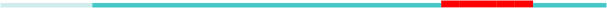
Obstructive sleep apnea	2.9	2014	2018	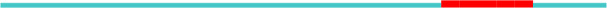
Alzheimers disease	3.41	2016	2018	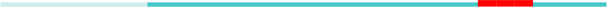
Nitric oxide	3.06	2016	2017	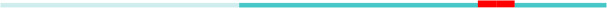
Memory impairment	2.93	2016	2022	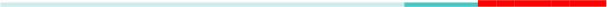
Spatial memory	2.97	2017	2019	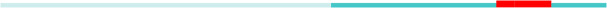
Reaction time	5.34	2018	2020	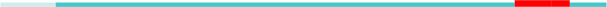
Mood	3.23	2018	2022	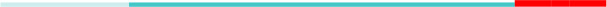

In addition, we placed keywords that appeared at least three times, plus their occurrence time and frequency on one coordinate to show the changes in development trends in recent years (Figure 7C). “Hypertension” was the most recent theme in this field, mainly referring to high-altitude-induced pulmonary hypertension. In recent years, scholars have focused on the mechanisms of brain injury in sleep disturbances and cognitive impairment at high altitudes, focusing on the cellular level. Furthermore, research on therapeutic tools such as acetazolamide and positive airway therapy were also hot subjects.

## 4 Discussion

Our visual analysis of global research on sleep disturbances and cognitive impairment at high altitudes in the period 1990–2022 showed an increasing trend in the output of publications. In addition, this increase showed exponential growth, indicating that research on sleep disturbances and cognitive impairment induced by high-altitude environments was still in the developmental stage, and that the trajectory to solve this problem will be bumpy and long. Moreover, high-altitude hypoxic environments also increase the complications of cardiovascular diseases, and studies on pulmonary hypertension are gradually increasing, and joint studies are the main trend in the future. This study not only added objective physiological data on sleep and nocturnal breathing at high altitudes, but also provided new perspectives and directions for altitude-induced sleep disturbances research.

### 4.1 Unique features and limitations

A total of 512 publications on sleep disturbances and cognitive impairment at high altitudes were initially retrieved, and 487 publications were eventually included for consideration. We decided to select all articles and reviews from 1990 to 2022 to ensure that a high number and quality of publications was included in the analysis and that our analysis was comprehensive enough to make reliable predictions about research hotspots. Scholars have recognized the exceptional quality of publications in the WOSCC SCI-Expanded database. We conducted a comprehensive visual analysis of the retrieved articles with the help of four packages of scientometric software, namely, R Bibliometrix, VOSviewer, CiteSpace 6.1.R6, and Microsoft Excel. We could comprehend the recent research interest in this topic by conducting a qualitative and quantitative study of the articles, analyzing the countries, affiliations, journals, authors, and citations of articles involved to identify the best contributors and contributions in the field. We sorted through the keywords of papers and assessed the co-citation, co-authorship, and co-occurrence relations among countries, affiliations, journals, authors, and keywords to gain a better sense of the main research directions and hot spots for this topic. This was the first bibliometric study to explore the development of high altitude-induced sleep disorders and cognitive impairment, giving it both reference and instructional significance.

However, our research still had some limitations. First, although the WOS Core Collection SCI-Expanded database included a large number of high-quality research publications, some literature may have been overlooked. Second, when analyzing information on authors, the Bibliometrix package does not necessarily categorize authors by their full names, such as only using initials for their names. For example, “Zhang Y” could be either “Zhang Yuan” or “Zhang Yang,” but Bibliometrix cannot distinguish between them, which will lead to inaccurate results. It is a flaw of the Bibliometrix package, so we used VOSviewer rather than the Bibliometrix package for the author information analysis. Furthermore, when analyzing keywords, it is natural to believe that when terms have a higher frequency, this indicates that research is more concentrated there. However, this may lead us to neglect some pivotal words with fewer appearances, especially when the field is emerging and developing.

### 4.2 Future research trends

Firstly, a number of basic and clinical observational studies have been conducted to explore the underlying brain pathology of sleep disturbances and cognitive impairment at high altitudes. The unique hypobaric hypoxia at high altitudes poses a major threat to the hippocampus, a part of the brain structure that plays a critical role in cognitive functions such as learning and memory (Titus et al., 2007). Extremely high-altitude exposure may cause white and gray matter changes affecting brain regions involved in motor activity, a severe problem for climbers (Di Paola et al., 2008). Titus et al. revealed that when exposed to a hypobaric hypoxic environment, both the CA1 and CA3 regions of rat hippocampal pyramidal neurons exhibited neuronal fixation, neuronal degeneration, and apoptosis, which may be the primary reasons for impaired stability of neural circuits and memory dysfunction in the hippocampal region (Maiti et al., 2007). Unexpectedly, the dendritic length and spine number of pyramidal neurons in the CA1 region increased significantly after 21 days’ exposure to hypobaric hypoxia, and the spatial memory ability of rats improved (Maiti et al., 2008a). This recovery may be related to prefrontal cortex plasticity and hippocampal pyramidal neurons’ dendritic plasticity (Maiti et al., 2008a). Although rats are resistant to hypoxic damage, the human brain may be more severely affected under the same altitude conditions. Therefore, we need to consider the differences between the species when researching other clinical diseases and plateau physiological abnormalities when encountering hypoxic ischemia in the brain. The plasticity and specific repair mechanisms of human hippocampal neurons under long-term acute or chronic hypoxic conditions need further investigation.

Secondly, sleep disturbances and cognitive impairment at high altitudes interact and reinforce each other. High-altitude hypoxia causes brain function and neurological damage by affecting structures such as the hippocampus and cortex; on the other hand, it has important effects on cognitive function through physiological factors such as sleep disturbance. High-altitude hypobaric hypoxia induces respiratory abnormalities in the body, resulting in frequent awakenings and inadequate perception of air during sleep, reducing total sleep time and slow-wave sleep, which in turn leads to inefficient sleep (San et al., 2013). Clinical manifestations include hypoventilation, periodic breathing, obstructive sleep apnea, central sleep apnea, etc. In this condition, emotions such as depression, frustration, anxiety, and anger cannot be suppressed and human cognitive function becomes impaired. This is reflected in the inability to concentrate, reduced ability to work, and gradual deterioration of memory and executive skills (San et al., 2013). Recently, scholars have increasingly focused on the relationship between high-altitude pulmonary hypertension (HAPH) and high-altitude-induced sleep disturbances, cognitive impairment, and other high-altitude diseases. Akylbek et al. proposed that hypoxia and other plateau-induced pathological alterations may underlie and/or contribute to HAPH (Sydykov et al., 2021). This may provide new ideas for research on the pathological mechanisms and the development of new targets for various high-altitude diseases.

Thirdly, in recent years, research on the pathophysiological mechanisms of hypobaric hypoxia-induced sleep disturbances and cognitive impairment have attracted widespread attention. Oxidative stress and neuroinflammation in sleep disturbances and cognitive impairment induced by altitude hypoxia have been a focus of scholars’ attention since 2007. Under hypoxia conditions, the body’s metabolic pathways are altered and oxidative phosphorylation is inhibited, resulting in the production of large quantities of superoxide anions, hydrogen peroxide, hydroxyl radicals, peroxynitrites, and nitric oxide (NO) (Wang X. et al., 2022). In this case, the explosion of free radicals directly damages the basement membrane of the blood–brain barrier, triggering vasogenic edema. In addition to directly producing oxidative stress responses, ROS also act on microglia and astrocytes to promote the production of pro-inflammatory and inflammatory cytokines such as interleukin-1β (IL-1β), IL-6, IL-1α, and tumor necrosis factor-α (Wang X. et al., 2022). These inflammatory cytokines induce dysregulation of blood–brain barrier transporters, damage the extracellular matrix and neurovascular units, and thus accelerate leukocyte migration and glial cell activation, leading to disruption of the brain microenvironment. Chronic poor sleep quality may lead to excessive activation of the hypothalamic–pituitary–adrenergic (HPA) axis and sympathetic pathways, promoting increased inflammatory cytokine activity, and ultimately leading to dysregulation of the inflammatory system, triggering neuroinflammation and emotional disturbances (Palagini et al., 2022). These responses triggered by neuroinflammatory factors may ultimately alter synaptic plasticity and lead to cognitive impairment (Wang X. et al., 2022). Studies have found that the development of sleep disturbances was associated with the activation of the canine uric acid pathway, degradation of tryptophan metabolism, and emotional severity (Mukherjee et al., 2018). In studies related to hypoxia-induced cognitive impairment, special mention has been made of the mTOR signaling pathway, the alterations *in vivo* have been associated with various neurological disorders. mTOR is normally regulated in acute hypoxic environments, but in chronic long-term hypoxic environments, mTOR is over-activated, leading to neuronal cell death with severe effects on the cognitive domain (Abdo Qaid et al., 2021). ATP5B, COX5A, GAPDH NDUFV2, SOD1, UQCRC1, and UQCRC2 have been reported as sleep deprivation and sleep disorder-related genes, and these studies have contributed to the development of candidate biomarkers for the diagnosis and treatment of plateau-induced sleep disorders (Abdo Qaid et al., 2021; Chen et al., 2021). The mechanisms underlying the onset and development of altitude-induced sleep disturbances and cognitive impairment are complicated, and sleep disturbances and cognitive impairment are interactive and mutually reinforcing. Therefore, it is a future research trend to explore the critical mechanisms of sleep disturbances and cognitive impairment at high altitudes and to find novel effective therapeutic targets.

Last, but not least, specific therapies for sleep disturbances and cognitive impairment at high altitudes are still in development. Presently, positive airway pressure therapy is used to reduce high-altitude sleep disturbances, improve sleep quality, and delay the deterioration of neurocognitive function by reducing the over-recruitment of brain regions (Jiang et al., 2021). However, a precise quantitative assessment of its effects is still lacking. Effective drugs targeting neurological dysfunction during high-altitude exposure have rarely been reported. During high-altitude exposure, comatose drugs such as amphetamines and dextroamphetamine have been found to improve psychomotor performance, but they severely disrupt sleep in a dose-dependent manner (Wang et al., 2013). Dexamethasone has also been reported to have a positive effect on high-altitude-induced cognitive impairment, but it may have side effects similar to systemic glucocorticoids (Wang et al., 2013). The effectiveness of acetazolamide in acute altitude sicknesses has been well documented, but more research is needed on the effectiveness of altitude cognitive impairment (Fischer et al., 2004). In this case, the exploration of other promising therapeutic agents is imminent. Acetylcholinesterase inhibitor, which is involved in preventing apoptosis-induced neuronal damage, can improve learning memory impairment in rats in an altitude oxygen chamber (Muthuraju et al., 2009). Ceftriaxone also rescued hippocampal neurons from excitotoxicity and promoted recovery from hypoxia-induced memory impairment on the plateau (Hota et al., 2008). Furthermore, herbal medicines such as *Bacopa monniera* and quercetin have been confirmed to have neuroprotective effects and having potential as drugs to improve spatial memory impairment on the plateau (Hota et al., 2009; Liu et al., 2015). In the future, there is an urgent need to translate these fundamental research results into clinical applications and to develop new, effective clinical interventions for patients with plateau sleep disturbances and cognitive impairment.

## 5 Conclusion

From 1990 to 2022, the number of publications on sleep disturbances and cognitive impairment at high altitudes was generally on the rise. Research in this area is of high medical value and is still in the developmental phase. We analyzed the scientific production in this field from different countries, the largest contributor being the United States. The institution with an outstanding contribution was the University Hospital Zurich. Bloch Conrad E. was the most valuable author in this field, having the most publications and citations, and his research was of high academic significance. The journal with the most publications in this area was High Altitude Medicine and Biology, and the most cited was Aerospace Medicine and Human Performance. Both have high professional recognition and abroad academic influence. In recent years, High Altitude Medicine and Biology has become a more popular choice for scholars to publish their research. The results of keyword analysis suggested that the research hotspots were focused on the clinical manifestations of sleep disturbances and cognitive impairment caused by altitude hypoxia, including “acute mountain-sickness,” “insomnia,” “apnea syndrome ,” “depression,” “anxiety,” ''Cheyne-strokes respiration,” and “pulmonary hypertension,” and on the mechanisms of cognitive impairment at high altitudes mainly associated with “oxidative stress,” “inflammation,” “hippocampus,” “prefrontal cortex,” “neurodegeneration” and “spatial memory” in the brain.

As far as we know, our article is the first systematic literature analysis on sleep disturbances and cognitive impairment at high altitudes. The results of our analysis will help scholars to better understand the global developments and changing trends in this field.

## Data Availability

The original contributions presented in the study are included in the article/supplementary material, further inquiries can be directed to the corresponding author.
